# Bioinspired Soft Machines: Engineering Nature’s Grace into Future Innovations

**DOI:** 10.3390/jfb16050158

**Published:** 2025-04-28

**Authors:** Ajay Vikram Singh, Mohammad Hasan Dad Ansari, Arindam K. Dey, Peter Laux, Shailesh Kumar Samal, Paolo Malgaretti, Soumya Ranjan Mohapatra, Madleen Busse, Mrutyunjay Suar, Veronica Tisato, Donato Gemmati

**Affiliations:** 1 Department of Chemical and Product Safety, German Federal Institute for Risk Assessment (BfR), Max-Dohrn-Straße 8-10, 10589 Berlin, Germany; 2 The BioRobotics Institute, Scuola Superiore Sant’Anna, 56025 Pontedera, Italy; 3 Department of Excellence in Robotics & AI, Scuola Superiore Sant’Anna, 56025 Pontedera, Italy; 4 STEMAZE, 69126 Heidelberg, Germany; 5 Unit of Immunology and Chronic Disease, Institute of Environmental Medicine, Karolinska Institute, 17177 Stockholm, Sweden; 6 Centre for Applied Research in Data Science, Indian Institute of Technology-Ropar, Ropar 14001, India; 7 Helmholtz-Institut Erlangen-Nürnberg for Renewable Energy (IET-2), Forschungszentrum Jülich, Cauerstr.1, 91058 Erlangen, Germany; 8 School of Biotechnology, KIIT University, Bhubaneswar 751024, India; 9 Federal Institute for Risk Assessment (BfR), Department of Biological Safety, Diedersdorfer Weg 1, 12277 Berlin, Germany; 10 Department of Translational Medicine, University of Ferrara, 44121 Ferrara, Italy; 11 Centre Hemostasis & Thrombosis, University of Ferrara, 44121 Ferrara, Italy

**Keywords:** bioinspired materials, adaptive materials, nature-inspired design, soft machines, adaptive robotics

## Abstract

This article explores the transformative advances in soft machines, where biology, materials science, and engineering have converged. We discuss the remarkable adaptability and versatility of soft machines, whose designs draw inspiration from nature’s elegant solutions. From the intricate movements of octopus tentacles to the resilience of an elephant’s trunk, nature provides a wealth of inspiration for designing robots capable of navigating complex environments with grace and efficiency. Central to this advancement is the ongoing research into bioinspired materials, which serve as the building blocks for creating soft machines with lifelike behaviors and adaptive capabilities. By fostering collaboration and innovation, we can unlock new possibilities in soft machines, shaping a future where robots seamlessly integrate into and interact with the natural world, offering solutions to humanity’s most pressing challenges.

## 1. Introduction to Bioinspired Soft Machines

**Figure 1:**
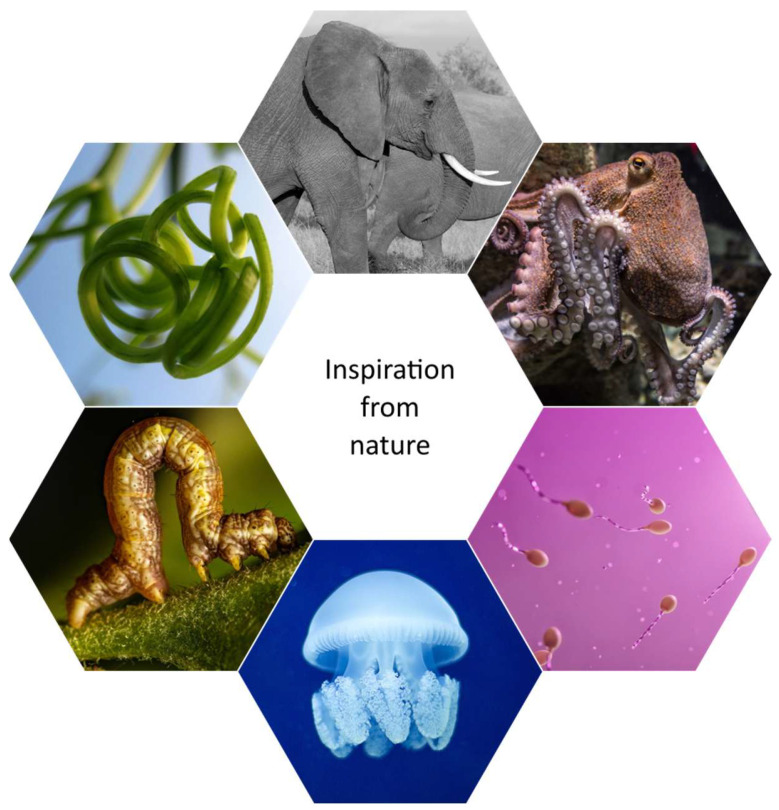
Nature-inspired robotics motion: Various biomimetic motions inspired by nature, including inchworm locomotion, jellyfish propulsion, earthworm peristalsis, octopus tentacle movements, sperm motility, elephant trunk manipulation, and earthworm burrowing. These bioinspired motions serve as inspiration for the design and development of robotic systems capable of navigating diverse environments with adaptability and efficiency (figure designed with biorender.com).

Soft machines are robotic systems primarily composed of compliant, deformable materials that enable them to safely adapt to unstructured environments and interact safely with humans and biological tissue. Soft machines stand at the forefront of technological innovation, aiming to replicate the remarkable capabilities of biological systems in artificial machines. By mimicking the flexibility and adaptability observed in nature, soft machines have the potential to revolutionize various industries and applications [1]. Bioinspired materials play a key role in this field, offering unique properties that enable robots to interact safely and effectively with their environment. The significance of bioinspired materials in soft machines lies in their ability to infuse robots with characteristics parallel to living organisms [2]. These materials are carefully selected to mimic the mechanical properties of biological tissues, allowing for soft machines to move with agility, conform to different surfaces, and interact delicately with objects and living beings [3,4]. Through bioinspired design principles, researchers aim to create robots that can navigate complex environments with ease and perform tasks that were once reserved for living creatures [5]. The three most important characteristics of soft machines are their flexibility, adaptability, and resilience in their motion, enabling them to navigate complex environments, interact safely with humans, and perform delicate tasks with precision [6], inspired by different scale body parts in the animal kingdom such as those shown in Figure 1 [7].

## 2. Understanding Biological Inspiration

**Figure 2:**
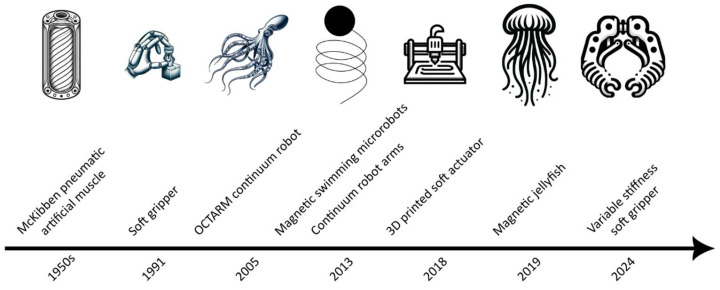
Evolution of soft machines: This figure illustrates the transformative journey of soft machines, from its conceptualization as an interdisciplinary field merging biology, materials science, and engineering to the development of robots inspired by nature’s elegant solutions (figure designed with biorender.com).

Nature has long served as a source of inspiration for human innovation, and in the realm of soft machines, the study of biological systems has proven particularly fruitful. One of the most captivating aspects of biology is how animals exploit soft structures to navigate their environments with remarkable proficiency and adaptability [8]. Understanding these biological inspirations not only sheds light on the fine details of natural systems but also provides valuable insights for the design and development of soft robotic technologies [9]. As shown in Figure 2, the timeline for bioinspired soft machines encompasses the evolution of soft machines from inception, highlighting key milestones where interdisciplinary efforts in biology, materials science, and engineering have converged to shape the field [10]. Through exploration of nature’s elegant solutions and ongoing advancements in bioinspired materials, the narrative traces the trajectory towards designing robots that mimic the adaptability and versatility found in natural organisms. This timeline underscores the collaborative efforts driving innovation in soft machines, leading to robots integrating into complex environments, having the potential to offer transformative solutions to societal challenges.

Animals ranging from cephalopods to mammals leverage the inherent flexibility and dexterity of soft structures to achieve a diverse array of movements and tasks. For instance, the octopus is a creature renowned for its ability to contort its body and manipulate objects with astonishing agility. The octopus achieves such feats through the coordination of its soft, muscular tentacles, which exhibit a level of flexibility and responsiveness unparalleled in the realm of robotics [11]. By mimicking the mechanics of these tentacles, engineers have developed soft robotic arms capable of navigating confined spaces and delicately manipulating objects with a finesse reminiscent of their biological counterparts [12].

Similarly, the elephant provides another compelling example of nature’s ingenuity in soft locomotion. Despite its massive size, the elephant possesses a trunk endowed with a remarkable degree of flexibility and strength. This multifunctional trunk serves as both a sensory organ and a versatile tool for grasping and manipulating objects in its environment [13]. By emulating the structural properties and functionalities of the elephant trunk, researchers have created soft robotic grippers capable of gripping objects of various shapes and sizes with remarkable precision and efficiency [14,15].

These examples underscore the remarkable adaptability and efficiency of soft structures in biological systems, providing inspiration for the design and development of innovative soft robotic technologies. By studying and emulating nature’s solutions to complex challenges, researchers can unlock new avenues for enhancing the capabilities of soft machines in a wide range of applications, from healthcare to manufacturing and exploration [16]. The survey of biological inspirations for soft machines offers a wealth of insights into the remarkable capabilities of natural systems. By drawing inspiration from creatures such as the octopus and the elephant, researchers can develop soft robotic technologies that not only emulate the agility and adaptability of their biological counterparts but also push the boundaries of what is possible in the field of robotics. Our understanding of biology will continue to grow, and so will our ability to harness nature’s ingenuity to drive innovation in soft machines [17].

## 3. Materials Selection in Soft Machines

The choice of materials is paramount in the design and development of soft machines, as it directly influences their performance, functionality, and potential applications [18,19]. Unlike traditional rigid robots, soft machines rely on pliable materials that can deform and adapt to their environment, enabling them to navigate complex and dynamic settings with ease [20]. While material selection also depends on parameters like cost, weight, and manufacturability, in this section, only the physical properties of the materials used in soft machines are explored, highlighting various types of soft materials and discussing key considerations for material properties.

**Figure 3:**
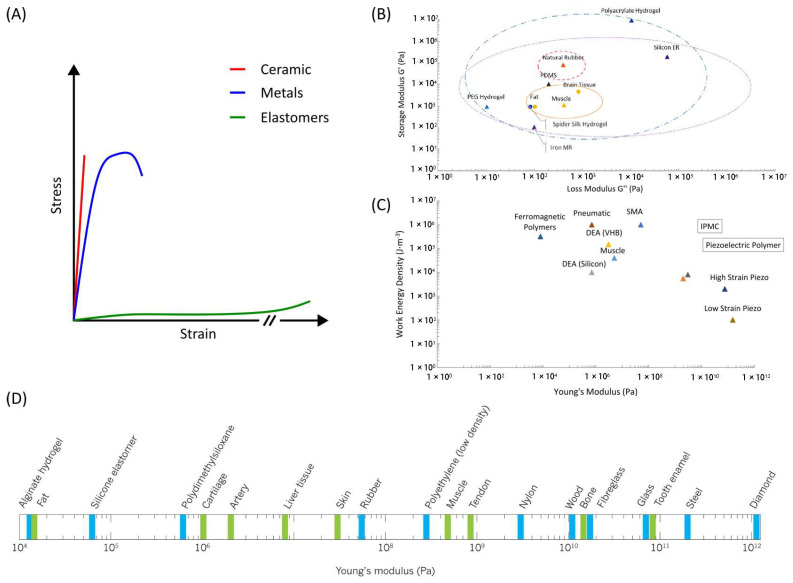
Mechanical characteristics of soft materials in soft machines: (**A**) Typical stress–strain curve plots depicting the mechanical behavior of various materials commonly used in soft machines, such as silicone elastomers, urethanes, hydrogels, braided fabrics, hydraulic fluids, and gases. Materials like ceramics and metals have an elastic modulus much higher than soft materials like polymers and elastomers. On the other hand, polymers and especially elastomers can withstand much higher strains and deform accordingly, making them more compliant. Composites composed of a combination of any of these materials would have properties somewhere in between. (**B**) Approximation of storage modulus vs. loss modulus of various organic and inorganic materials. Hydrogels (blue line); biological tissue (yellow line); natural rubber (red line); electrorheological (ER) and magnetorheological (MR) fluid-based polymers (purple line). Materials that have been used in soft machines: triangle; hard materials: diamond. (**C**) Work energy density vs. Young’s modulus of established soft materials. Initialisms: SMA—shape memory alloy; IPMC—ionic polymer-metal composite; DEA—dielectric elastomer. Reprinted with permission from Ref. [26]. Copyright 2018, Elsevier Ltd. (**D**) Understanding the Young’s modulus of these soft components is crucial for designing and fabricating soft machines with the desired mechanical properties, including flexibility, resilience, and responsiveness to external stimuli. Soft machines are composed primarily of materials with moduli comparable with those of soft biological materials, which not only makes them compliant, but also leads to compliance matching. Reprinted with permission [18]. Copyright 2015, Springer Nature Limited.

Unlike rigid materials commonly used in traditional robotics, soft materials possess unique mechanical properties such as flexibility, elasticity, and compliance, allowing for soft machines to interact safely and effectively with their surroundings, as depicted in Figure 3 [21]. The choice of materials influences not only the mechanical behavior of soft machines but also factors such as weight, cost, and manufacturability [22]. A diverse array of soft materials is available for use in soft machines, each offering distinct advantages and limitations. Elastomers, such as silicone rubbers and polyurethanes, are commonly used in soft machines due to their excellent elasticity and durability [23]. For example, Ecoflex (Smooth-On) can stretch more than 500% before breaking, whereas hard materials like steel typically have a yield strain of less than 2.5% [24]. These materials can undergo large deformations without permanent damage, making them well-suited for applications requiring compliant and stretchable structures, including actuators and artificial skins [25] (Figure 3A).

Hydrogels represent another class of soft materials with unique properties, including high water content and tunable mechanical properties [27]. Hydrogels exhibit similarities to biological tissues, making them suitable for applications such as biomedical devices and soft actuators [28]. Shape memory polymers, on the other hand, offer the ability to recover their original shape upon exposure to external stimuli, enabling programmable and reversible deformations in soft robotic systems [29]. When selecting materials for soft machines, several key considerations must be taken into account to ensure optimal performance and functionality [30]. Flexibility is a critical property, allowing for soft machines to deform and conform to their environment while maintaining structural integrity (Figure 3A). Choosing viscoelastic materials allows for soft machines to dissipate energy and maintain stable motion during dynamical loading. The viscoelastic behavior is often described in terms of the storage and loss moduli (Figure 3B). A higher loss modulus (compared to virtually no loss modulus for purely elastic material like steel) indicates the material’s ability to dissipate energy. Durability is also essential, particularly in applications subject to repeated or prolonged mechanical stresses [31]. Another important metric is the work energy density of materials. The higher the energy density of the actuator, the less volume needed to meet the design objective. There seems to be a trade-off between stiffness with the energy density of materials as seen in Figure 3C. Stiffer materials tend to have higher energy densities but are less compliant. Therefore, the right material choice depends on the application. To achieve compliance matching, materials with a similar Young’s modulus to a soft biological material can be considered (Figure 3D). For example, the Young’s modulus of polydimethylsiloxane (PDMS) (~1 MPa) is similar to that of cartilage [32,33]. While magnetic soft composites can also show a similar Young’s modulus to that of cartilage [34], their properties are dependent on their composition. For example, higher concentrations of magnetic particles can increase the stiffness and Young’s modulus. Similarly, phenomena like magnetostriction or magnetoelastic stiffening can also alter the mechanical behavior of magnetic composites in the presence of a magnetic field [35]. Biological cells and tissues can also be used directly as materials for soft machines [36,37]. Considerations such as biocompatibility are crucial for applications involving interaction with biological systems, ensuring compatibility and safety [38]. Therefore, materials selection plays a central role in the design and development of soft machines, influencing their mechanical behavior, functionality, and potential applications [39]. Table 1 gives key example properties and aspects of bioinspired materials found in the literature.

Considering factors such as flexibility, durability, and biocompatibility, the unique properties of soft materials can be harnessed to create innovative and versatile soft robotic systems capable of addressing a wide range of challenges across various domains [45]. Table 2 further exemplifies the practical application of these principles, showcasing a range of bioinspired products currently available on the market. This selection highlights the diverse and innovative ways in which bioinspired materials are being utilized across various industries.

**Table 2:** Examples of commercially available bioinspired products and their applications.

Company/Group Name	Bioinspired Theme	Product Name	Usage	Reference
Fusion Bionic	Nano-scale surface texture	Bioinspired nanotexture	Surface applications in various fields like medical and aerospace	[46]
GreenPod Labs	Plant-based volatiles	Packaging sachets	Sustainable packaging	[47]
Intropic Materials	Enzymatic processes	Plastic degradation	Plastic waste management	[48]
Biohm	Biomimicry	Circular construction	Sustainable construction	[49]
Terrapin Bright Green	Biomimicry in transportation	Biomimicry-inspired transportation solutions	Sustainable transportation systems	[50]
TISSIUM	Gecko adhesion	Surgical adhesive	Medical surgeries (tissue reconstruction)	[51]
SoftGripping	Soft grippers	GorillaFingers	Pick and place	[52]

## 4. Actuation Mechanisms in Bioinspired Soft Machines

### 4.1. Actuation Mechanisms

Soft machines, inspired by the capabilities of biological systems, have witnessed significant advancements in actuation mechanisms, enabling robots to exhibit lifelike movements and behaviors [53]. In this section, an overview of different actuation methods used in soft machines is provided, bioinspired actuation mechanisms are examined, and the advantages and challenges associated with each approach are discussed.

**Figure 4:**
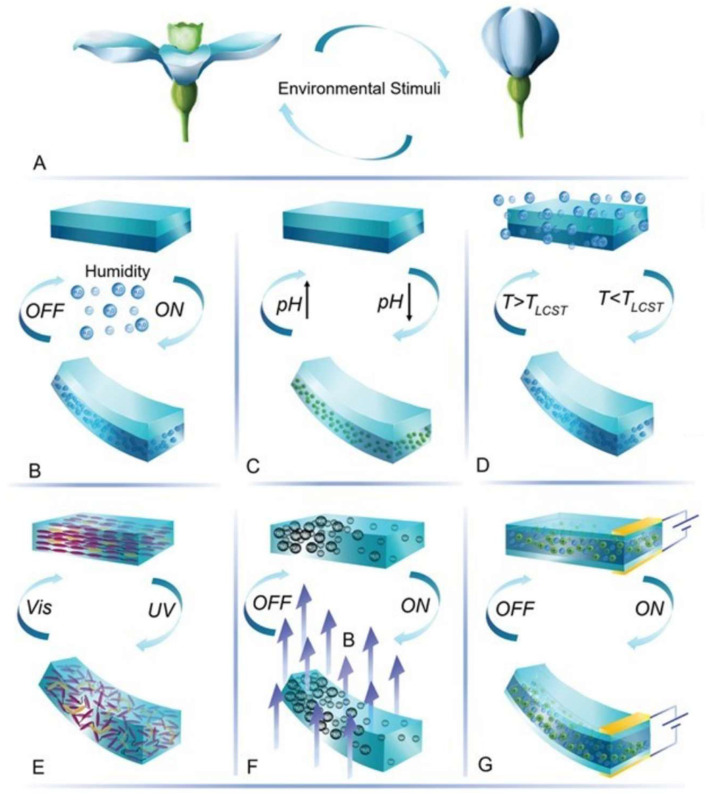
Stimuli-responsive soft machines inspired by plant motion: This figure illustrates stimuli-responsive soft machines inspired by plant motion, incorporating (clockwise, peripherally) hydraulic, pneumatic, electrical, and chemical stimuli-responsive mechanisms. (Centre) Mimicking the adaptive responses observed in plants to (**A**) environmental cues, these soft machines demonstrate versatile motion capabilities, enabling them to navigate and interact with their surroundings effectively. The integration of diverse stimuli-responsive systems: (**B**) humidity, (**C**) pH, (**D**) temperature, (**E**) light, (**F**) magnetic fields, and (**G**) electrical fields enhance the adaptability and functionality of these robots, promising advancements in fields such as agriculture, environmental monitoring, and soft machine research (Figure designed with Microsoft Designer & biorender.com).

**Figure 5:**
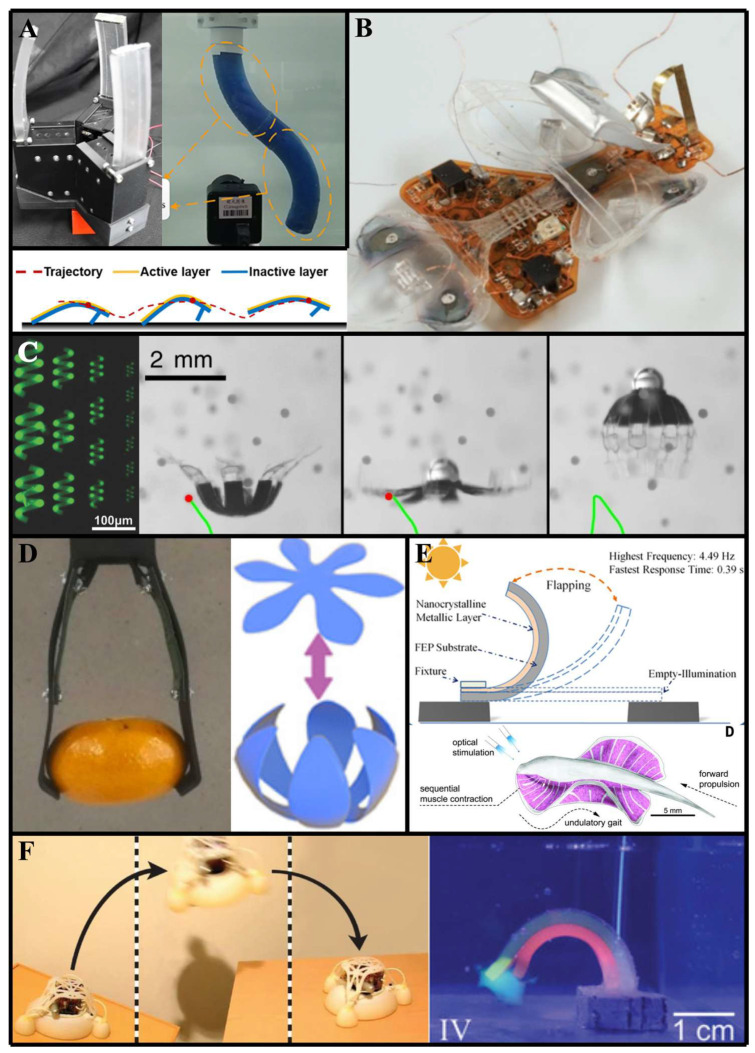
Examples of common actuation mechanisms used in bioinspired soft machines: (**A**) Fluidic (pneumatic or hydraulic) (Adapted from Ref. [57]; Reprinted with permission from Ref. [58]. Copyright 2020, IOP Publishing). (**B**) Electrical (Reprinted with permission from Ref. [62]. Copyright 2019, American Association for the Advancement of Science; Reprinted with permission from Ref. [63]. Copyright 2019, American Association for the Advancement of Science). (**C**) Magnetic (Reprinted from Ref. [67]; Reprinted with permission from Ref. [68]. Copyright 2018, Wiley). (**D**) Thermal (Reprinted from Ref. [70]; Reprinted with permission from Ref [71]. Copyright 2016, ACS Publications). (**E**) Light (Adapted with permission from Ref. [72]. Copyright 2020, ACS Publications. Reprinted with permission from Ref [73]. Copyright 2016, American Association for the Advancement of Science). (**F**) Chemical (Reprinted with permission from Ref. [75]. Copyright 2023, Wiley. Reprinted with permission from Ref [76]. Copyright 2015, American Association for the Advancement of Science).

Soft machines utilize a variety of stimuli or actuation methods (Figure 4), each offering unique advantages and challenges:

(a) Pneumatic actuation relies on the use of pressurized air or gas to deform soft structures, enabling smooth and versatile movements [54]. Additionally, pneumatic networks represent a bioinspired approach to actuation, mimicking the distributed network of muscles and tendons found in biological organisms. By embedding channels within soft structures and selectively pressurizing them, soft machines can achieve complex movements and deformations, reminiscent of natural locomotion [55]. Hydraulic actuation, similar in principle to pneumatic actuation, uses pressurized liquid instead of gas, offering increased power and precision in certain applications [56]. Applications include grippers mimicking octopus tentacles (Figure 5A), where selective pressurization replicates muscular hydrostat dynamics [57,58].
(b) Electric actuation, on the other hand, involves the use of electrically driven components such as shape memory alloys or electroactive polymers to induce deformations in soft materials, providing precise control and responsiveness [59]. Shape memory alloys (SMAs), such as NiTi, exhibit a martensitic–austenitic phase transformation when heated, leading to substantial contraction forces and repeatable shape recovery [60]. Electroactive polymers (EAPs), including dielectric elastomers and ionic polymer–metal composites, deform under electric fields via Maxwell stress or ion migration, offering large strain outputs and muscle-like actuation profiles [61]. Applications include small-scale actuators with a fast response [62,63] (Figure 5B).
(c) Muscle-like actuators, for example, emulate the contractile properties of biological muscles, enabling soft machines to exhibit dynamic and adaptive movements [64]. These actuators can be fabricated using materials such as dielectric elastomers or pneumatic artificial muscles, offering a high degree of compliance and controllability [65].
(d) Magnetic actuation involves the use of magnetic fields and gradients to manipulate soft composites embedded with ferromagnetic or superparamagnetic particles. Hard magnetic materials enable programmed deformation through spatially varying magnetization profiles, while soft magnetic materials exhibit torques and forces due to field-induced magnetization, enabling rapid, wireless, and untethered actuation with complex spatiotemporal control [34,66]. Applications include µm or mm scale remotely actuated robots with multiple functionalities [67,68] (Figure 5C).
(e) Thermally responsive actuation employs materials that undergo volumetric or mechanical transitions upon heating, triggered via infrared (IR) or near-infrared (NIR) radiation, thermal conduction, or Joule heating through conductive networks. Examples include hydrogels, thermoplastic elastomers, and liquid crystal elastomers, where thermal input modulates phase behavior, stiffness, or swelling, resulting in controlled morphological transformations [69,70,71] (Figure 5D).
(f) Light-responsive materials are based on photochromic molecules that capture optical signals and convert them into different property modifications. Applications include biomimetic insects and fishes, which need high spatial and temporal control for actuation [72,73] (Figure 5E).
(g) Chemical-based actuators utilize different chemical reactions to either produce energy or phase change that actuates the robot [74]. Applications include autonomous soft robots and high-energy actuations [75,76] (Figure 5F).

Each actuation method in soft machines offers distinct advantages and faces unique challenges. Pneumatic actuation, for instance, provides excellent compliance and adaptability but may suffer from limited power and response time. Hydraulic actuation offers increased power and precision but may be constrained by the complexity of fluid management systems. Electric actuation offers high precision and controllability, particularly beneficial for tasks requiring fine manipulation. EAPs and SMAs can be electrically stimulated to produce complex, programmable deformations while being compact. However, these actuators often require high driving voltages (in the case of dielectric elastomers) or efficient thermal management systems (as with SMAs), along with sophisticated power electronics, and encapsulation to prevent electrical hazards [77]. Magnetic actuation provides remote actuation abilities and excellent controllability but suffers from low actuation forces [78]. Magnetic actuation enables wireless, remote control, making it ideal for minimally invasive environments, such as in vivo biomedical/robotic applications. Its response time is typically fast, and the direction and magnitude of actuation can be modulated spatially and temporally. Nonetheless, magnetic actuation generally suffers from limited force and torque output, particularly at small scales and over larger distances, due to spatial attenuation of magnetic fields and gradients [78]. Moreover, the requirement for external magnetic sources or field-generating systems can constrain the portability and scalability of the system [79]. Thermally responsive actuation provides an intrinsically safe and stimulus-specific mechanism, suitable for autonomous or environmentally triggered actuation. Materials such as liquid crystal elastomers or hydrogels with critical transition temperatures can be tailored for specific thermal responses. However, thermal actuation tends to exhibit slower response and recovery times due to heat transfer limitations and material thermal inertia, making it less suitable for applications demanding rapid cyclic actuation. Additionally, continuous heating can pose challenges for energy efficiency and material durability over extended operation [69]. Light actuation is advantageous in terms of its remote nature, but it requires a direct line of sight with the actuator, limiting its use to surface applications. Chemical actuation can provide strong actuation forces but suffers from low controllability [74].

Bioinspired actuation mechanisms offer the potential for soft machines to emulate natural movements and behaviors convincingly, but they also present challenges in terms of scalability, efficiency, and control. Overcoming these challenges requires interdisciplinary collaboration and innovative approaches that leverage insights from biology, materials science, and engineering [80]. By drawing inspiration from biological systems and exploring innovative actuation methods, researchers and engineers can unlock new possibilities for soft machines, enabling robots to exhibit lifelike movements and behaviors in a wide range of applications [81].

### 4.2. Control Strategies

While soft robots’ continuum deformation is an advantage in making them compliant, it also introduces infinite degrees of freedom (DoFs), complicating traditional control frameworks. Some control strategies used for soft robots include the following:

(a) Reduced-order modeling: Finite element methods (FEM) approximate soft body dynamics. For instance, Ye et al. modeled octopus-inspired arms using Cosserat rod theory, reducing computational load by 70% [11].
(b) Proprioceptive feedback: Stretchable sensors can be used to enable real-time strain mapping. For example, the SoftSCREEN colonoscope uses curvature feedback for closed-loop navigation [4]. Sujit et al. demonstrated inductive sensing for precise, low-hysteresis strain measurement and closed-loop control in soft robots [82]. Polykretis et al. demonstrated adaptive neural network-based control of DEAs [83].
(c) Feedforward control: Simple repetitive tasks like soft gripper actuation can be performed using preprogrammed pressure sequences [84].
(d) Embodied intelligence: Control can be offloaded to material properties. For example, the continuous deformability of octopus-inspired arms is utilized for complex manipulation [85].

## 5. Embodied Intelligence in Bioinspired Soft Machines

While reduced-order models and proprioceptive feedback enable more efficient simulation and real-time control of soft robots, they often rely on external computation or complex calibration. Feedforward control, though simple and effective for repetitive tasks, cannot handle disturbances in unstructured environments. Embodied intelligence, by contrast, exploits the robot’s material properties and morphology to perform complex tasks with minimal sensing and control. This makes it especially well suited for soft robots, whose compliant bodies can passively adapt to their surroundings, reducing the need for high-fidelity models or extensive sensor networks. Embodied intelligence is the concept that intelligent behavior arises not just from the control system, but also from the interaction between the body, the environment, and the control architecture. It emphasizes that the morphology of a system—its materials, shape, and mechanical properties—can contribute significantly to its ability to interact with and adapt to the world, often reducing the need for complex computation.

Embodied intelligence lies at the heart of bioinspired soft machines, leveraging the inherent properties of soft materials to achieve adaptive and versatile behaviors [86,87,88,89]. In this section, the concept of embodied intelligence, its relevance in soft machines, and the unique contributions of soft materials to this paradigm shift are discussed. Furthermore, the advantages of embodied intelligence over traditional algorithmic approaches in robotics are highlighted.

**Figure 6:**
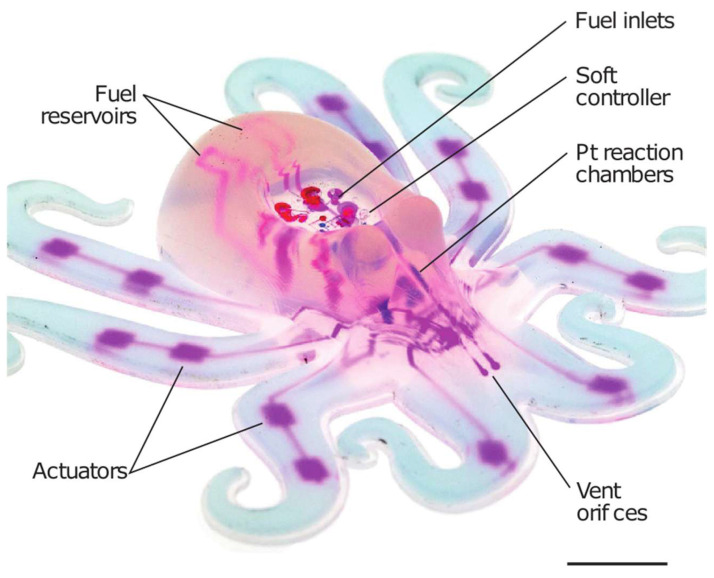
Soft materials and embodied intelligence in soft machines inspired by octopi and realized with microfluidic actuation: This figure highlights the contributions, relevance, and advantages of soft materials in embodied intelligence within soft machines, using the octopus as a prime example. Soft materials enable robots to achieve complex and adaptable movements, akin to the dexterity and versatility observed in the octopus. This illustration underscores the pivotal role of soft materials in enhancing the functionality and efficiency of soft robotic systems, paving the way for innovative applications across various domains. Reprinted with permission from Ref. [89]. Copyright 2016, Springer Nature Limited. Scale bar: 10 mm.

Embodied intelligence refers to the integration of perception, action, and cognition within the physical body of a robotic system, enabling it to interact with its environment in a dynamic and adaptive manner [26,90]. Traditional robots often rely on centralized control architectures and explicit programming to execute tasks, which can be computationally intensive and brittle in the face of uncertainty and variability. In contrast, embodied intelligent systems leverage the intrinsic capabilities of their bodies and the principles of self-organization to generate robust and adaptive behaviors without the need for explicit instructions. Unlike traditional robots, which rely heavily on preprogrammed algorithms to execute specific tasks, embodied intelligent systems derive their behaviors from the interactions between their bodies, sensors, and surroundings. This approach mirrors the decentralized and emergent nature of intelligence observed in biological organisms, allowing for soft machines to exhibit lifelike movements and behaviors without the need for complex computation [91,92]. It also allows for soft machines to navigate unpredictable environments, interact with complex objects, and learn from experience in a manner that closely resembles biological organisms [93,94]. Therefore, the intrinsic properties of soft materials play a crucial role in enabling embodied intelligence in bioinspired soft machines [95]. The integration of embodied intelligence simplifies principles for robot control by focusing on control parameters, albeit introducing complexities in robot design [96]. As shown in Figure 6, by exploiting these properties, soft machines can exhibit a form of “embodied cognition”, where their physical bodies serve as a substrate for intelligence, enabling them to perceive, process, and respond to sensory stimuli in real time [97]. In all such examples, soft bodyware is essential for obtaining emergent behaviors from external interactions, highlighting the interconnected nature of embodied intelligence and soft machines [96].

The main advantage of soft materials for embodied intelligence in soft machines is the adaptability. Soft machines with embodied intelligence can think autonomously based on sensory inputs, enabling human-like interactions with the environment and enhancing adaptability in complex and changing geometries. By mimicking the neural networks of living organisms, these robots can exhibit complex behaviors, learn from interactions, and adapt to changing conditions autonomously [83]. Embodied intelligence allows for soft machines to navigate and interact with their environment more naturally, making them suitable for tasks like assisting surgeons or search and rescue operations in disaster areas. They excel in adapting and responding to dynamic environments, offering advantages over rigid robotic systems that struggle in complex or changing terrains [94].

Embodied intelligence represents a paradigm shift in robotics, where the physical properties of soft materials are harnessed to imbue robots with adaptive and versatile behaviors. As shown in Table 3, soft machines are much better equipped for adaptability and compliance compared to conventional hard robots. The continued exploration of embodied intelligence holds promise for the development of soft machine technologies that can seamlessly integrate into and interact with the natural world, opening up new possibilities for applications in fields such as healthcare, exploration, and beyond.

## 6. Applications and Future Directions of Bioinspired Soft Machines

Bioinspired soft machines have emerged as a ground-breaking field with diverse applications, promising transformative advancements across various domains [98]. An overview of current applications is provided in this section, potential future directions and advancements are discussed, ethical considerations and societal impacts associated with bioinspired soft machines are also addressed.

Bioinspired soft machines hold great promise for addressing a wide range of challenges and advancing technological innovation [99,100,101,102,103,104]. By leveraging insights from biology and embracing interdisciplinary collaboration, researchers and engineers can continue to push the boundaries of what is possible in soft machines, unlocking new opportunities for applications and addressing ethical considerations and societal impacts along the way. As we embark on this journey towards a future shaped by bioinspired soft machines, it is imperative to remain mindful of our responsibilities and ensure that these technologies serve the common good and contribute to the well-being of society [32,105].

### 6.1. Current Applications

**Figure 7:**
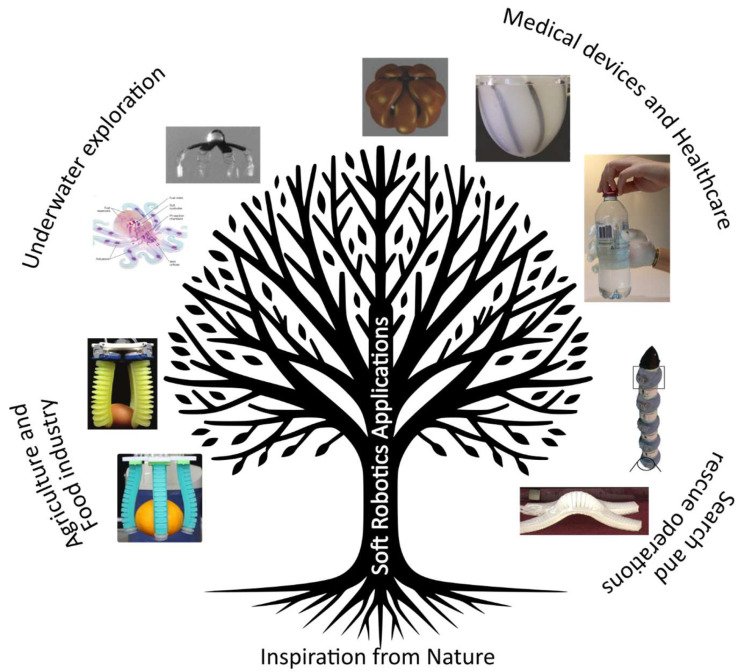
Nature-inspired soft robot applications: This figure depicts the diverse applications of nature-inspired soft machines across agriculture, underwater exploration, medical health, and search and rescue operations. Examples include agricultural robots mimicking human hands for delicate crop handling (Adapted with permission from Ref. [99]. Copyright 2020, Elsevier Ltd.; Adapted with permission from Ref [100]. Copyright 2018, Springer Nature Limited), underwater robots inspired by jellyfish and octopus propulsion for efficient marine exploration (Adapted with permission from Ref. [89]. Copyright 2016, Springer Nature Limited; Adapted from Ref [67]), soft medical robots replicating the movement of muscles for minimally invasive surgeries (Adapted with permission from Ref [101]. Copyright 2014, Wiley. Adapted with permission from [81]. Copyright 2014, Wiley; Adapted from Ref [102].), and resilient soft machines inspired by snakes and worms for navigating complex terrain during search and rescue missions (Adapted from Ref. [103]; Adapted from Ref [104]).

As shown in Figure 7, bioinspired soft machines are already making significant contributions to fields such as healthcare, search and rescue, and exploration [106]. In healthcare, soft machines offer minimally invasive solutions for surgical procedures, enabling precise and gentle interventions with reduced risk to patients [107,108]. Soft machines are being explored for applications such as targeted drug delivery and prosthetics due to their flexibility and biocompatibility [109]. Soft robotic exosuits and prosthetics provide personalized assistance and rehabilitation for individuals with mobility impairments, enhancing their quality of life and independence [110]. In search and rescue operations, bioinspired soft machines can navigate confined spaces and complex terrain with agility, aiding in disaster response efforts and saving lives [111]. Their adaptability and ability to navigate complex terrains can be crucial for locating and assisting individuals in disaster scenarios [109]. In environmental exploration, bioinspired soft machines can navigate challenging terrains, monitor ecosystems, and collect data in ways that traditional rigid robots cannot. Their flexibility and adaptability make them ideal for exploring remote or hazardous environments [112]. Furthermore, soft robotic grippers and manipulators find applications in manufacturing and assembly, offering flexible and adaptable solutions for handling delicate objects and performing intricate tasks [113].

### 6.2. Future Directions and Advancements

The future of bioinspired soft machines holds immense potential for further innovation and advancement. One promising direction is the development of autonomous soft machines capable of self-repair and self-reconfiguration, enabling adaptive responses to changing environments and unforeseen challenges [114]. Integrating advanced sensing and artificial intelligence technologies into soft machines will enhance their perception, decision-making, and interaction capabilities, paving the way for intelligent and autonomous systems with human-like cognition [115]. Additionally, advances in materials science and fabrication techniques will enable the creation of soft machines with enhanced performance, durability, and biocompatibility, opening up new possibilities for applications in healthcare, wearable technology, and human–robot interaction [116]. For example, the integration of self-healing materials in soft machines is a groundbreaking innovation that enables robots to repair damage autonomously. Inspired by biological systems that can regenerate and heal, self-healing soft machines have the potential to enhance durability and longevity in various applications [117,118].

**Figure 8:**
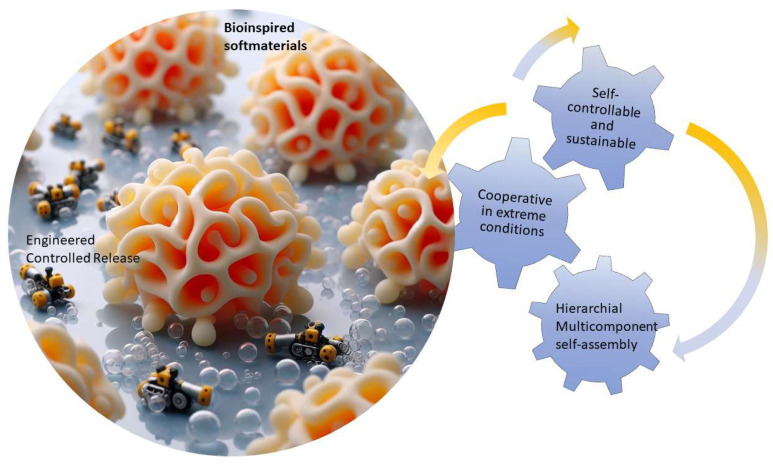
Soft robotic swarm for targeted drug release: Self-controllable small-scale robots that can adapt and navigate a complex path to reach the target area and give a coordinated response for a specific task, for example, controlled drug release. If needed, self-assembly could be performed to improve the effectiveness of a coordinated response. AI-assisted tools were used for initial conceptualization of this figure, but final designs were refined by the authors to reflect original research data (Figure designed with biorender.com).

These futuristic machines can also be autonomous, sustainable bioinspired robots that can mimic artificial organs. Non-experts could effortlessly utilize these machines capable of computing, sensing, and moving autonomously using embodied intelligence. A potential example is depicted in Figure 8, where self-controllable small-scale robots cooperate and, if needed, perform a self-assembly to give a coordinated response for a specific task, for example, controlled drug release in a difficult-to-reach place in the human body. These robots have the potential to outperform traditional counterparts, harness energy sustainably, and operate effectively in challenging environments [109,119]. As soft robotic technologies mature, there is a growing trend towards commercialization. Concepts developed in academia are transitioning into commercial enterprises, broadening the adoption of soft robotic systems across industries like food processing and industrial automation [109,120].

This convergence between materials and machines is a paradigm shift, where the soft body integrates all components necessary for actuation, sensing, and computation, while the “soft brain” handles software aspects, controlling the device and reasoning about the world and task goals [93,121]. To realize their true potential, extreme body compliance is essential, enabling tasks like inspecting pipes with complex geometries or performing laparoscopic surgery. Moreover, soft machines must contend with uncertainty, navigating rocky terrains or grasping unknown objects. For autonomy, proprioceptive localization is crucial, enabling soft machines to operate independently. Utilizing biologically inspired artificial intelligence, these soft machines promise to revolutionize various industries like healthcare and exploration by offering adaptive, versatile, and user-friendly solutions [121].

While soft machines hold immense potential, there are also some challenges. Biodegradation, especially in applications involving implantable machines, must be controlled to match its lifespan. The formation of bacterial biofilm on soft surfaces in moist environments due to their high surface area could lead to a risk of infection or machine failure [122]. Incorporating antimicrobial coating or developing self-cleaning surfaces could help overcome this challenge [123]. Precise control is another challenge for soft machines due to the compliant nature of the materials [124]. Addressing these challenges is crucial for their use in real-world applications.

## 7. Ethical Considerations and Societal Impacts

**Figure 9:**
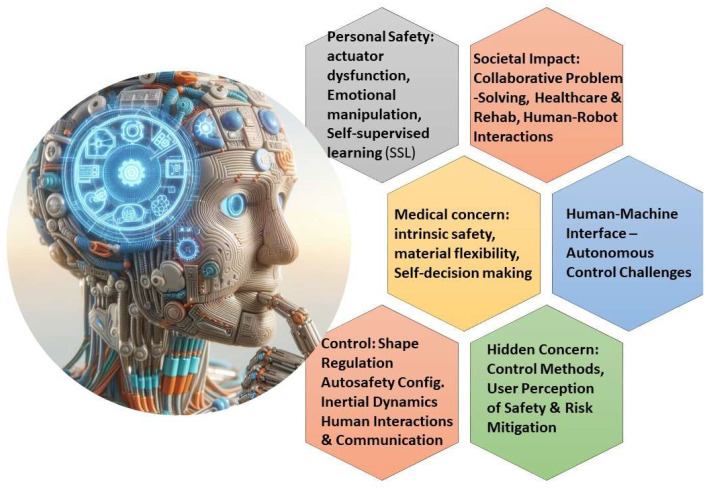
Ethical framework for bioinspired soft machines: The establishment of an ethical framework can guide the development and deployment of bioinspired soft machines. Addressing concerns including privacy, safety, and societal impact, this framework can ensure responsible innovation and adoption of these technologies, fostering ethical considerations throughout their lifecycle. AI-assisted tools were used for initial conceptualization of this figure, but final designs were refined by the authors to reflect original research data (Figure designed with Microsoft Designer & biorender.com).

As bioinspired soft machines continue to evolve, it is essential to address ethical considerations and societal impacts associated with their development and deployment. Ethical considerations include issues such as privacy, autonomy, and safety, particularly in applications involving human–robot interaction and healthcare, as shown in Figure 9 [125]. Ensuring transparency and accountability in the design, development, and deployment of soft machines is crucial to mitigate potential risks and ensure ethical standards are upheld [126]. Furthermore, addressing societal impacts involves considerations such as job displacement, economic inequality, and access to technology, as the widespread adoption of soft machines may reshape labor markets and social dynamics. Collaborative efforts involving stakeholders from diverse backgrounds will be essential to navigate these ethical and societal challenges and ensure that bioinspired soft machines benefits humanity as a whole [127]. Projects like the Horizon2020 EU initiative focusing on soft actuated heart development highlight the importance of addressing ethical concerns in medical applications [109,128]. Biohybrid systems that combine living cells or tissues with synthetic materials to open up new possibilities for applications in healthcare, environmental monitoring, etc., raise other ethical concerns [129,130]. Another ethical concern could come with the development of soft machines to function as artificial organs, which could offer solutions to millions of people awaiting transplants worldwide [109,131]. The potential for soft machines to contribute to artificial organs is significant given the global shortage of organ donors.

In the ever-evolving landscape of technology and safety standards, the integration of artificial intelligence (AI), machine learning (ML), and quantitative structure–activity relationship (QSAR) techniques help in the evaluation of material safety for soft machines [132,133]. This forward-looking approach not only ensures meticulous scrutiny of potential hazards but also enables swift identification and mitigation of risks. Moreover, the expanding field of digitalized toxicology adds another layer of sophistication to safety analysis methodologies, empowering stakeholders to proactively address safety concerns with precision and efficiency [134]. As we embrace these new approach methodologies (NAMs), the future of ensuring the safety of soft machines is propelled towards a realm of heightened reliability and transparency, safeguarding both users and the wider community [135,136].

## 8. Conclusions

The journey through the realm of bioinspired soft machines has unveiled a world of innovation, where the convergence of biology, materials science, and engineering has given rise to transformative technologies. The adaptability and versatility of soft machines, inspired by the elegant solutions found in nature, has been highlighted. By harnessing the intrinsic properties of soft materials, researchers have unlocked new possibilities for creating robots that integrate into and interact with the natural world. These materials, with their unique mechanical properties and functionalities, serve as the building blocks for creating soft machines with adaptive capabilities. By continuing to advance this field, new materials and fabrication techniques can be developed, enabling breakthroughs in healthcare, exploration, manufacturing, and beyond. A wide range of actuation principles have been developed for powering soft machines. Taking inspiration from nature, embodied intelligence has also been integrated into soft machines for a decentralized and autonomous decision-making. Several application scenarios have been found in the state of the art, and many more that can be developed as the field progresses have been discussed. Some ethical considerations when dealing with soft machines and efforts to tackle them have also been mentioned.

Cross-disciplinary collaboration between researchers, engineers, biologists, and other stakeholders is crucial for bridging the gap between biology and technology, facilitating the exchange of ideas and expertise, and accelerating the pace of innovation. Moreover, advancing innovation will push the boundaries of what is possible in soft machines and unlock new opportunities for addressing societal challenges.

Bioinspired soft machines offer a glimpse into a future where robots blend with the natural world, offering solutions to some of humanity’s most pressing challenges. By continuing to explore nature, harnessing the potential of bioinspired materials, and taking a multi-disciplinary route, we can unlock new possibilities for advancing soft machines and shaping a future where bioinspired robots will be used for the most crucial and needed applications.

## Figures and Tables

**Table 1:** Comprehensive table outlining key example properties and aspects of bioinspired materials for soft machines. While these values have been taken from the literature, they can change depending on the composition, testing conditions, and deformation methods.

Material Type	E	$\sigma_{UTS}$	$\epsilon_{f}$	Material Selection: Application	Actuation Criteria: Application	References
Electroactive polymers	0.01–10 MPa		>200%	Flexibility, responsiveness, durability	Electrical stimulation, mechanical deformation	[40]
Magnetic soft composites	~0.1–10 MPa	~2 MPa	>200%	Magnetic responsiveness, structural integrity	Magnetic fields	[34,35]
Stimuli-responsive hydrogels	10–100 kPa	1 kPa–1 MPa	2–100%	Swelling behavior, mechanical properties	Various stimuli (e.g., temperature, pH)	[41]
Liquid crystal elastomers	0.1–100 MPa	1–10 MPa	>200%	Mechanical properties	Various stimuli (e.g., heat, light, electricity)	[42]
Shape memory alloys	50–100 GPa	Up to 1.5 GPa	Up to 15%	Shape recovery, biocompatibility	Thermal activation	[43,44]

**Table 3:** Embodied intelligence: Comparison of the capabilities of soft machines due to their embodied intelligence versus conventional hard robots. Authors’ opinion about how advantageous they are in terms of different characteristics is indicated using a star system where ⋆ is the lowest; ⋆⋆ is moderate and ⋆⋆⋆ is the highest.

Robot Characteristics	Soft Machines	Conventional Hard Robotics
Compliance	⋆⋆⋆ Able to bend and twist with high curvatures and exhibit unprecedented adaptation, sensitivity, and agility. Soft materials are elastic and can deform and absorb much of the energy arising from a collision, so large degrees of freedom (DoFs).	⋆ Poor grasping power and mobility over soft surfaces. Hard materials perform single tasks efficiently, but often with limited compliance due to rigid links and joints.
Adaptability	⋆⋆⋆ Soft machines can adapt their shape to the environment, enabling their use in confined spaces.	⋆ Hard robots have limited adaptability due to rigid links and joints, restricting their use in confined spaces.
Material’s Young’s modulus	⋆⋆⋆ Soft materials like skin or muscle tissue have a Young’s modulus ranging from 10^4^ to 10^9^ Pa.	⋆⋆ Hard materials like metals or hard plastics have a Young’s modulus ranging from 10^9^ to 10^12^ Pa.
Actuation force	⋆ Soft structures are usually able to apply weak forces and torques.	⋆⋆⋆ Conventional actuators can apply high forces and torques.
Ease of integrating subsystems	⋆ Integrating sensing, actuation, computation, power storage, and communication into controllable soft-bodied material is difficult. Subsystems may move with respect to each other.	⋆⋆⋆ Subsystems can be attached firmly to the body.
Ease of fabrication	⋆⋆ Soft machines are usually fabricated using multimaterial 3D printing, soft lithography, and molding and casting.	⋆⋆⋆ Hard robots are usually fabricated using 3D printing, machining, and injection molding.
Ease of control	⋆ Soft machines have an infinite number of degrees of freedom due to their ability to bend, twist, stretch, compress, buckle, wrinkle, and exhibit elasticity. Control is challenging and requires new approaches to modeling, control, dynamics, and high-level planning.	⋆⋆⋆ Hard robots generally have 6 degrees of freedom (DoFs) (three rotations and three translations about the x, y, and z axes).
Actuation principle	⋆⋆⋆ Soft machines utilize fluidic, electrical, light-based, magnetic, chemical, or thermal actuation.	⋆⋆⋆ Hard robots usually utilize electric or fluidic actuation.
Sensing	⋆⋆ Soft machines use piezoelectric polymers, stretchable electronics, and various strains, including tensile, shear, or curvature, measured with layered channel geometries for sensing environmental signals.	⋆⋆⋆ Hard robots use encoders, metal or semiconductor strain gauges, or inertial measurement units (IMUs) for sensing.

## Data Availability

No new data were created or analyzed in this study. Data sharing is not applicable to this article.

## Abbreviations

The following abbreviations are used in this manuscript:

ER	electrorheological
MR	magnetorheological
SMA	shape memory alloy
IPMC	ionic polymer–metal composite
DEA	dielectric elastomer
AI	artificial intelligence
E	elastic modulus
$\sigma_{UTS}$	ultimate tensile strength
$\epsilon_{f}$	strain at failure
ML	machine learning
QSAR	quantitative structure–activity relationship
NAM	new approach methodology
